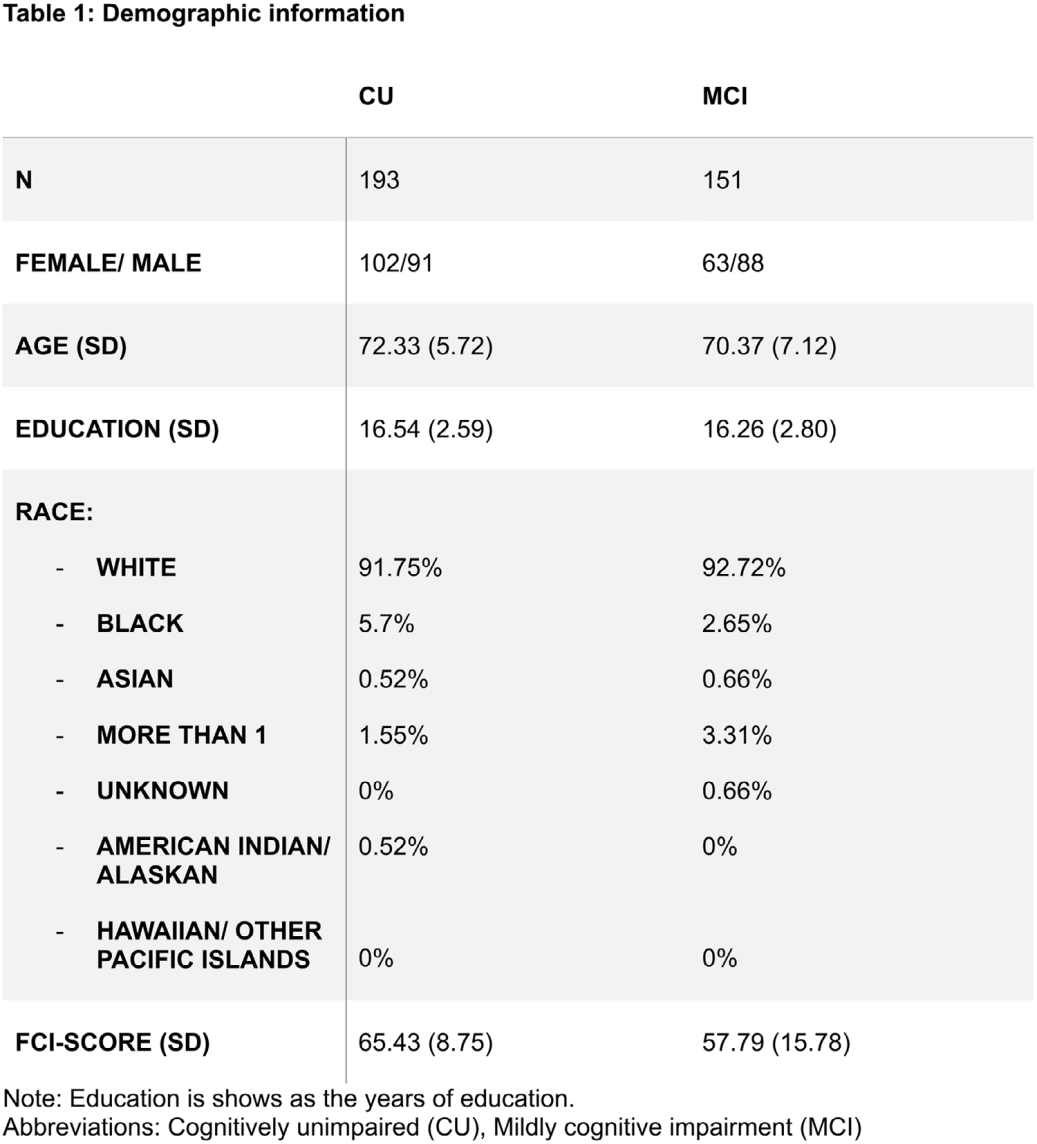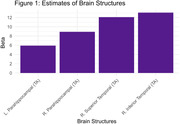# Neurocognitive Predictors of Financial Capacity: Exploring Commonalities in Brain Structure and Predictive Value of MRI in Pre‐Dementia Stages of Alzheimer’s Disease

**DOI:** 10.1002/alz.093120

**Published:** 2025-01-09

**Authors:** Kayla Le, Andrei Bieger, Thomas Hugentobler Schlickmann, Marco De Bastiani, Sarah Wehle Gehres, Wyllians Vendramini Borelli, Eduardo R. Zimmer, ADNI

**Affiliations:** ^1^ University of Applied Sciences Hamm‐ Lippstadt, Hamm, North Rhine‐ Westphalia Germany; ^2^ Universidade Federal do Rio Grande do Sul, Porto Alegre, Rio Grande do Sul Brazil; ^3^ Federal University of Rio Grande do Sul, Porto Alegre, Rio Grande do Sul Brazil; ^4^ Alzheimer’s Disease Neuroimaging Initiative, http://adni.loni.usc.edu/, CA USA

## Abstract

**Background:**

Several studies have shown that financial capacity constitutes a vital component of instrumental activities of daily living. However, there is insufficient research investigating the relationship between financial impairment, brain volume changes and cognitive decline in Alzheimer’s disease (AD). Here, we examine the association between brain volume changes and financial capacity in cognitively unimpaired (CU) and mild cognitively impaired (MCI) individuals.

**Method:**

A total of 344 individuals who had completed the Financial Capacity Instrument‐ Short Form (FCI‐ SF) assessment and underwent magnetic resonance imaging (MRI) imaging from the Alzheimer’s Disease Neuroimaging Initiative (ADNI) were selected. Volume and cortical thickness average were extracted from the MRI images. **Table 1** reports demographic information. Linear regression analysis explored associations between the structural brain features and the total FCI‐Score. The model was adjusted for age, gender, education and race. Lastly, stepwise regression analysis was performed to determine the predictors of financial capacity.

**Result:**

MCI individuals performed significantly worse on the FCI‐SF compared to CU individuals (p < 0.01, Cohen’s d = ‐ 0.55). Our model indicates that the temporal lobe and parahippocampus are bilaterally associated with the FCI‐Score (Figure 1). Stepwise regression analysis revealed that left parahippocampus thickness (p < 0.05) is a significant predictor for the FCI Score (Figure 1).

**Conclusion:**

Our results are consistent with earlier research showing changes in financial capacity in pre‐dementia stages, emphasizing important brain structures with potential predictive character. These findings enhance our comprehension of how brain structural changes are linked to deficits in financial skills before dementia and further evidence to consider these as early warning signs for cognitive decline.